# Implications of conflict on vaccination in the Sahel region

**DOI:** 10.1136/bmjgh-2024-016496

**Published:** 2025-01-30

**Authors:** Majdi M Sabahelzain, Harriet Dwyer, Seye Abimbola, Julie Leask

**Affiliations:** 1 The University of Sydney School of Public Health, Sydney, New South Wales, Australia; 2 Public Health, Ahfad University for Women School of Health Sciences, Omdurman, Khartoum, Sudan; 3 Department of Global Health and Development, London School of Hygiene and Tropical Medicine, London, UK

**Keywords:** Immunisation, Vaccines, Measles, Poliomyelitis, Global Health

## Abstract

The Sahel region is a geographical belt in Africa that stretches from the Atlantic Ocean to the Red Sea, between the Sahara Desert in the north and the Savannah in the south. It is characterised by challenging environmental crises and conflicts. This analysis highlights the potential implications of conflict on vaccination across five Sahel countries, including Burkina Faso, Chad, Mali, Niger and Sudan, from 2019 to 2023. It also presents recommendations to improve vaccination coverage in these settings. The WHO Immunisation Data Portal was used to extract data about vaccination coverage and disease outbreaks. With the increasing complexity of humanitarian access in the Sahel, there has been an accumulation of the number of zero-dose and underimmunised children. In 2023 alone, most of these countries had a significant proportion of zero-dose children, particularly Sudan (43%), Mali (22%) and Chad (16%). Nearly half of children in Sudan (49%), 33% in Chad and 23% in Mali are underimmunised. Measles vaccine coverage was consistently below 90% in these countries, except for Burkina Faso. The trend of polio outbreaks (circulating vaccine-derived poliovirus) across these countries showed fluctuations in the number of cases, with Niger having reported several cases over this period, and Chad having 101 cases reported in 2020 alone. Despite relatively high coverage, there were significant outbreaks of polio in Burkina Faso, Sudan and Mali in 2020, which reflects the potential impact of the COVID-19 pandemic. Lessons can be learnt from past diplomatic and programmatic successes, while investments in innovative and flexible approaches may help increase the reach of vaccination programmes in inaccessible areas.

**Summary box**

- The Sahel region is experiencing impacts of conflict on health systems and infrastructure alongside outbreaks of vaccine-preventable disease and concerning rates of zero-dose children.
- Local, regional and global health insecurity is evident from the emergence and circulation of genetically linked polio variants within the region. This may be attributed to the mobility and transnationalism of the population within the Sahel region, either as nomads, refugees or internally displaced populations including unvaccinated and undervaccinated populations.
- Providing vaccination and other health services in the Sahel region is challenging due to non-state armies controlling significant areas. Ministries of Health need to be supported by wider political processes drawing on broader diplomatic channels, new multilateral frameworks for vaccination in inaccessible areas and stronger funding for community-level infrastructure to engage with trusted intermediaries and political figures.
- The lack of real-time data and the poor quality of the available data on vaccine-preventable diseases make it difficult to understand the real situation in the Sahel. Greater investment in improved data collection and analysis, and innovations to address data gaps are urgently needed to inform and tailor effective vaccination interventions.
- The region needs innovative approaches to overcome the context-specific threats caused by instability, including using advanced methods such as geographical information systems, exploring co-delivery of vaccinations, and investing in building community and political trust in vaccines.

## Introduction

 Over the last two decades, the Sahel region in Africa (the Sahel) has experienced drought, famine and desertification as a consequence of climate change. Amid these environmental crises, security challenges in the region have become increasingly complex. Simmering localised ethnic conflicts have escalated alongside the widespread presence of light weapons, the growth of organised crime and criminal networks, and the emergence of extremist armed groups.^1^

**Figure 1:**
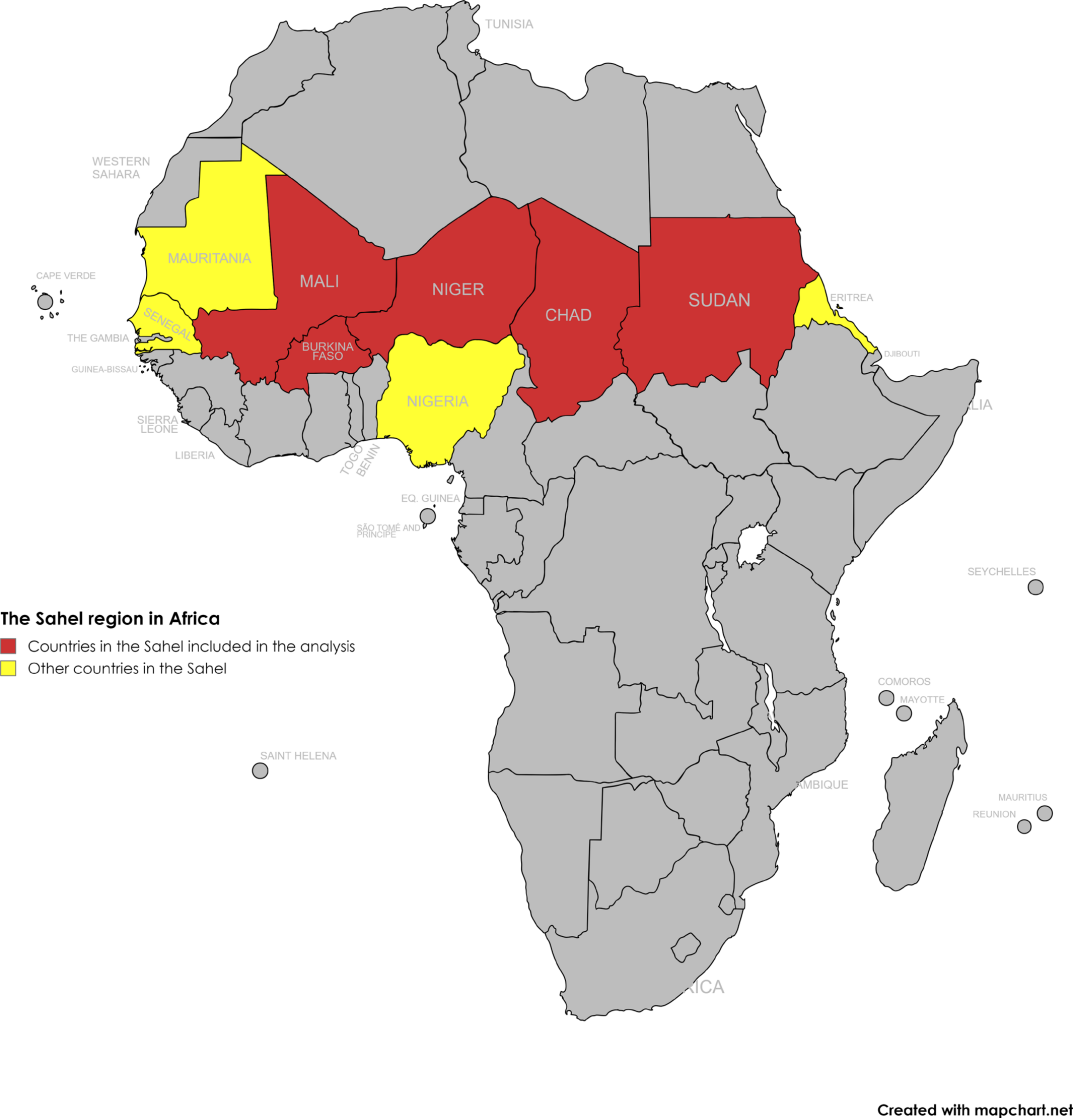
The Sahel region in Africa.

The Sahel is a vast geographical area with a semi-arid climate that spans several countries. Appearing on maps as a belt that stretches from Mauritania and Senegal in the west, all the way to Sudan and Eritrea in the east, the region is situated between the Sahara Desert in the north and the Savannah in the south (figure 1).^2^ Culturally and historically, *Sahel* is a word of Arabic origin that means coast or shore. It is also defined in some literature as the southern edge of the Sahara Desert.^1^ This region represents an interface between the Middle East and sub-Saharan Africa, with Arabic, Islamic and nomadic cultures from the north, and indigenous and traditional cultures, including local languages and religions from the south. These factors make the Sahel a unique site not only for cultural exchange and economic interaction between nomadic pastoralists and sedentary farmers but also for ethno-religious tensions.^1^

This region holds significant natural resources that imply geopolitical importance and attract the economic interests of several high-income countries.^3^ The growing involvement of international external actors in the region has led to significant changes in the last 3 years. For example, French troops have withdrawn from central Sahel countries, including Burkina Faso, Mali and Niger; the United Nations peacekeeping mission in Mali has ended; and Russia has established a military and economic presence.^4^

The Immunisation Agenda 2030 (IA2030) is a global strategy for vaccines and immunisation for the decades 2021–2030.^5^ It recommends prioritising and targeting communities with zero-dose and underimmunised children. ‘Zero-dose’ means they have not received any dose of diphtheria, tetanus and pertussis (DTP) containing vaccine, while underimmunised means they have received less than the recommended three doses of the DTP-containing vaccine, which could be one or two doses.^6^ Given that in 2023, UNICEF reported that 40% of zero-dose children are in fragile and conflict-affected settings,^6^ the agenda places particular emphasis on mobile populations, including refugees, internally displaced people and nomadic communities, that represent a significant proportion of populations in the Sahelian countries.^1^

This article aims to analyse vaccination coverage across five Sahelian countries. The analysis includes rates of zero dose and underimmunised children, and vaccine-preventable disease (VPD) outbreaks, including measles, along with circulating vaccine-derived poliovirus (cVDPV) from 2019 to 2022. These countries include Burkina Faso, Chad, Niger, Mali and Sudan, with estimated populations (in millions) in 2024 of 23.8, 18.8, 28.2, 24 and 49.4, respectively.^7^ These five countries are currently experiencing various forms of crisis. This article highlights the occurrence of conflict and instability while also providing an analysis of vaccination rates in these states which can be used here as a proxy for gaps in other health services.

The WHO Immunisation Data Portal was used to extract data about vaccination coverage and VPD outbreaks from 2019 to 2023.^8^ This data portal contains global, regional and country summaries of VPD-reported cases and WHO/UNICEF Estimates of National Immunisation Coverage (WUENIC).

### Security context

The Sahel region has experienced increasing political instability in recent years, with a wave of coups d’état (becoming the so-called ‘coup belt’ or ‘Junta belt’).^9^ Junta is a term that refers to a government led by a group or committee of military leaders. Since 2021, military takeovers have occurred in Sudan, Mali, Chad, Burkina Faso and, most recently, Niger, with growing consequences for communities swept up in these changes.^10^

Insecurity in the central Sahel, in Burkina Faso, Mali and Niger is complex, with various non-state armed groups involved. The nature of conflict and insecurity is varied and cuts across the region unbound by current national borders.^1^ These include the tensions between nomadic communities and farmers, particularly in the tri-border region between Burkina Faso, Mali and Niger. Some are nationalist groups including those calling for an independent Tuareg State in northern Mali, while others are more influenced by pan-Islamic movements and claim to be part of either Al-Qaeda or the so-called Islamic State group.^2^

Conflict and insecurity have weakened central security forces and their control, resulting in significant territories in these five countries being completely outside the control of the central governments. For example, in Burkina Faso, it is estimated that Jihadist groups—non-state armed groups—control about 40% of the country’s territory, mostly in the north and near the border with Mali.^11^ While the Tuareg ethnic group seized control of much of north Mali in 2012, with sporadic fighting occurring ever since.^2^ In Sudan, since the outbreak of war in April 2023, the Rapid Support Forces have seized control of most territories of Khartoum state and western Sudan (Darfur states).^12^

### Challenges to providing humanitarian assistance

The Sahel belt is home to many vulnerable population groups including nomads (both Afro and Arab nomadic groups), refugees, internally displaced people and people living in territories outside of government control. These groups make up a significant proportion of the total population in each country.^12 13^,^17^

The complex security context continues to fuel mass displacement and surging humanitarian needs for a significant proportion of these vulnerable communities. In 2023, The United Nations Office for the Coordination of Humanitarian Affairs (OCHA) estimated that about 1 in 5 people in Chad, 1 in 3 in Burkina Faso, Mali and Niger, and 1 in 2 in Sudan were in need of urgent humanitarian assistance.^14^,^18^ At the same time, funding for humanitarian programmes in this region remains critically low.^18^

Insecurity and humanitarian crises fuel a breakdown—and sometimes deliberate destruction—of vital health services. Depleted human resources mean reduced or stalled outreach services in accessible areas or for populations that have crossed borders. At the same time, investments are not made in supporting effective partnerships with community-level civil society to drive outreach and engagement. This situation has been documented across the Sahel.^19^

#### Fragility of health systems

Existing fragile healthcare systems in more stable areas lack the resilience to meet growing humanitarian needs.^20^ As a result, they are unable to provide adequate essential health services, such as vaccination and nutrition, which increases population vulnerability to disease outbreaks including VPDs.^13^,^17^ The fragmentation of governance in these contexts has already contributed to inequitable access to health services in both stable and unstable territories.^12^

#### Attacks on health infrastructure

At the same time, humanitarian access is becoming increasingly complex and restricted, leaving vulnerable populations with limited access to vital assistance. Humanitarian organisations operating in active conflicts have been subjected to attacks, which have put their operations in danger or made them impossible. It is estimated that in 2020, one-third of global abductions of aid workers occurred in Mali, Niger and Burkina Faso.^21^ In Mali, there were reports of an exponential increase in attacks on humanitarian organisations, implying challenges to the delivery of assistance.^22^ In other countries in the region, members of humanitarian organisations have been abducted or killed, and their facilities and vehicles have been attacked and seized.^23^

#### Competing interests in negotiating humanitarian access

The competing interests of armed actors undermine the delivery of humanitarian assistance, hampering public health programmes. For example, in the Nuba Mountains in Sudan, which is controlled by the Sudan People’s Liberation Army/Movement-North (SPLA/M-N), there are tensions around the administration of humanitarian assistance. The federal government prefers to see humanitarian assistance coordinated through Khartoum, while SPLM-N wants this assistance to come through South Sudan or Ethiopia. Disagreements undermine humanitarian efforts, negatively affecting vulnerable populations in need of assistance, particularly displaced children who are often the most vulnerable.^24^

#### Challenges to delivering vaccines in areas controlled by non-state actors

In areas controlled by non-state actors, there are significant challenges to delivering vaccines. Compounding this is the compromised quality of vaccination data and the absence of population-specific analysis, tailored strategies, governance and guidance. This is due to the fact that these areas are inaccessible to governments and traditional vaccination organisations such as WHO and UNICEF.^25 26^

In areas controlled by non-state armed groups in Jebel Marra in Darfur, Sudan, for example, the Expanded Programme on Immunisation (EPI), collaborates with trusted non-governmental organisations (NGOs) such as Médecins Sans Frontières (MSF) and individuals on the side of the armed groups to deliver vaccines.^27^ MSF and Save the Children, along with other organisations, are critical providers of health services, including vaccination, in states such as Khartoum and three states in Darfur regions that are either fully or partially controlled by the paramilitary Rapid Support Forces. Vaccines are provided via the Ministry of Health to these organisations.^28^

In Burkina Faso, health services, including vaccination, have been disrupted due to regular terrorist attacks since 2015. To address this issue, resilience strategies were initiated in 2019. One of these strategies involved shifting the management of immunisation activities to community health workers (CHWs).^29^ The process began with the training of district teams, then head nurses and immunisation officers of selected health facilities, who then provided comprehensive training to CHWs. A single study evaluating this approach found that it led to improved vaccine coverage.^29^ One challenge in implementing local strategies, however, is evaluating their effectiveness, particularly in complex crisis contexts.^27^

#### Trust in vaccination in the region

In the context of conflict, insecurity and displacement, vaccination services can be further undermined by an absence of trust. Impacted by extreme conditions of uncertainty; trust processes shift where existing social structures have been frayed. At the same time, the trust chain is an important lever for vaccination acceptance.^30^

The 2003 polio vaccine boycott in the northern zones of Nigeria, for instance, demonstrates the effect of pervasive mistrust on relations between northern Nigerian communities, leaders and the West.^31^ Until recently, Nigeria was one of the three polio-endemic countries in the world—a problem that had stemmed from the boycott of the vaccine, which lasted 11 months.^31^ Islamic leaders boycotted the polio vaccination campaign, following a surge of political tensions due to political differences between the predominantly Muslim northern states and the southern presidential candidate who won. These tensions were further heightened by underlying geopolitical factors, such as 9/11 and the subsequent ‘war on terror’.’ Due to the perception that this war was aimed at Muslims, rumours spread that polio vaccinations were being used to infect people with HIV and render males sterile to reduce Muslim populations.^31^ Since then, the Boko Haram group, which was already established in 2002 in northern Nigeria, has been actively spreading these rumours and challenging the eradication of Polio in the region.^32 33^

In mid-2004, diplomatic efforts by different trusted parties played a crucial role in restoring trust in vaccines and resuming the programme in northern Nigeria and neighbouring regions in other countries including Niger and Chad. Representatives from northern Nigeria were sent to South Africa, Indonesia and India to verify the quality of the polio vaccine. As a result, they recommended Biopharma, an Indonesian company based in a Muslim country, to be the new supplier of polio vaccines for the northern states.^31^

Now, as the conflict in the northern zones of Nigeria and its borders with Niger, Chad and Cameroon continues to simmer and health systems remain fragile, the conditions are prime for again questioning vaccines and fomenting ongoing community suspicions about the motives behind vaccination programmes.^34^ Additionally, the re-emerging, splintering and rebranding of the Boko Haram group, which already pledged allegiance in 2015 to the self-proclaimed Islamic State and rebranded as the Islamic State in the West African Province,^35^ may have a negative impact on vaccination in other regions in the Sahel. This is particularly concerning given the control of large territories by other jihadist groups in the tri-border region between Burkina Faso, Mali and Niger.

### Vaccination in the Sahel against the backdrop of conflict

Globally 21 million children were classified as either ‘zero dose’ or underimmunised children in 2023. Furthermore, it is estimated that 6%–15% of these ‘zero-dose children’ are residing in conflict-affected settings.^36^ An analysis of vaccination coverage data from 16 conflict-affected countries, including three countries from the Sahel found that almost 67% of global polio cases and 39% of global measles cases from 2010 to 2015 were reported from these countries.^19^

In recognition of the impact of conflict settings on vaccination, Gavi, the Vaccine Alliance, launched the Zero-dose Immunisation Programme in 2022. The programme aims to address inequity in vaccinating the most vulnerable children in 11 countries in the Sahel and the Horn of Africa regions. The programme is managed by two consortia of organisations that operate in these complex settings, including RAISE 4 Sahel (Reaching and Adapting Immunization Services Effectively to Reach Zero-Dose Children in the Sahel) in Burkina Faso, Chad, Mali and Niger and REACH (Reaching Every Child in Humanitarian) in the Horn of Africa including Sudan.^37^

### The vaccination situation in the five countries in the Sahel

DTP and measles vaccinations are important milestones for children. Children receive three doses of DTP-containing vaccines in most countries within the first months of life. During vaccination sessions, oral polio vaccine (OPV) doses are administered along with DTP-containing vaccine. They also receive two doses of measles-containing vaccine in the first and second years. Gavi linked zero-dose and underimmunisation definitions with DTP vaccination for operational purposes,^38^ and it is used as a proxy for all routine childhood vaccinations. Other definitions have linked it to the non-receipt of any routine vaccination. Polio (cVDPV) and measles cases are used as indicators for the failure of first and second-milestone vaccinations. Table 1 contains coverage rates for DTP and measles-containing vaccines and the number of measles and polio cases.

**Table 1:** Coverage of DTP-containing vaccine and measles-containing vaccines and number of measles and polio cases*

Country	Indicators	2023	2022	2021	2020	2019
Burkina Faso	DTP1	99%	97%	95%	95%	95%
	DTP3	94%	93%	91%	91%	91%
	Number of polio cases (cVDPV)	3	–†	2	65	1
	MCV1	94%	91%	88%	88%	88%
	MCV2	71%	71%	71%	71%	71%
	Number of measles cases	680	252	–†	2480	672
Chad	DTP1	84%	82%	79%	74%	69%
	DTP3	67%	60%	58%	52%	50%
	Number of polio cases (cVDPV)	55	44	0	101	11
	MCV1	63%	54%	53%	45%	40%
	MCV2	35%	2%	–‡	–‡	–‡
	Number of measles cases	11 862	2158	2577	2170	1882
Mali	DTP1	78%	78%	79%	75%	81%
	DTP3	77%	77%	77%	70%	77%
	Number of polio cases (cVDPV)	15	2	0	52	0
	MCV1	73%	73%	72%	62%	71%
	MCV2	59%	44%	33%	26%	4%
	Number of measles cases	364	765	2074	498	454
Niger	DTP1	94%	96%	94%	93%	92%
	DTP3	85%	84%	82%	81%	81%
	Number of polio cases (cVDPV)	3	15	18	10	1
	MCV1	80%	65%	80%	79%	79%
	MCV2	68%	42%	66%	60%	58%
	Number of measles cases	1708	12 898	9271	2754	10 321
Sudan	DTP1	57%	75%	93%	98%	99%
	DTP3	51%	68%	84%	90%	93%
	Number of polio cases (cVDPV)	–†	1	0	59	0
	MCV1	51%	66%	81%	86%	90%
	MCV2	38%	51%	63%	68%	74%
	Number measles cases	1377	–†	–†	401	3555

* The vaccination coverage data are based on WUENIC estimates.

† No data available from the WHO dashboard.

‡ The vaccine was not introduced to the country.

cVDPVcirculating vaccine-derived poliovirusDTPdiphtheria, tetanus and pertussisMCVmeasles-containing vaccineWUENICWHO/UNICEF Estimates of National Immunisation Coverage

This analysis presents an overview of the available data on immunisation coverage and VPD outbreaks in five Sahelian countries. The data were extracted from the WHO data portal, which contains global, regional and country summaries of VPD-reported cases and WUENIC.

#### Challenges of vaccination coverage data in conflict settings

The estimates of vaccination coverage in the Sahel countries, as well as other low- and middle-income countries encounter major reliability issues. In part, due to the use of census projections to calculate the target children for vaccination each year. For instance, the last census conducted in Sudan and Chad was in 2008 and 2009, respectively.^39 40^ There are, also operational challenges that are specific to the Sahel region. A significant percentage of the population residing in these countries, for example, has been leading a life of constant mobility and transnationalism for a long time. This mobility can be due to their lifestyle, such as nomadic groups, or because they are refugees or internally displaced, or living in territories controlled by non-state armies. As a result, they are often left out of national censuses and surveys, making them invisible to the government and other organisations.^1^

The WUENIC estimate accounts for these biases in reported vaccination coverage data from many countries and, therefore, uses multiple sources, including administrative, official and survey coverage data, as well as published and grey literature.^41^

Acknowledging the challenges and limitations in estimating vaccination coverage and VPD cases reported in the WHO portal, we analyse the data as the official available information.

### Vaccination coverage

#### DTP vaccination coverage

For DTP vaccination, IA2030 has set a goal to reduce the number of zero-dose children by 50% by the year 2030.^6^ Additionally, it aims to achieve a global coverage rate of 90% for the DTP3 vaccine in order to control and eliminate these diseases. Burkina Faso and Niger have exhibited high coverage rates for the first dose of DTP-containing vaccine over a 5-year period. For example, in 2023, DTP1 coverages were 99% and 94%, respectively, with a low proportion of zero-dose children in the two countries. However, the vaccination coverages of DTP3 in all these countries, except Burkina Faso, have been suboptimal (≤85%), which suggests significant proportions of children are underimmunised in each country over these 5 years. For instance, in 2023, nearly half of the children in Sudan (49%), one-third in Chad (33%) and one-fourth in Mali (23%) did not receive a full course of DTP vaccines (three doses). In the same year, the proportion of zero-dose children ranged from 1% to 43% in these five countries with significant proportions in Sudan (43%), Mali (22%) and Chad (16%).

The accumulation of the number of zero-dose and underimmunised children over a 5-year period across these five countries reveals the persistent risk of polio, diphtheria, pertussis tetanus and other VPDs (of which vaccines are usually administered during the same vaccination sessions).

#### MCV coverage

**Figure 2:**
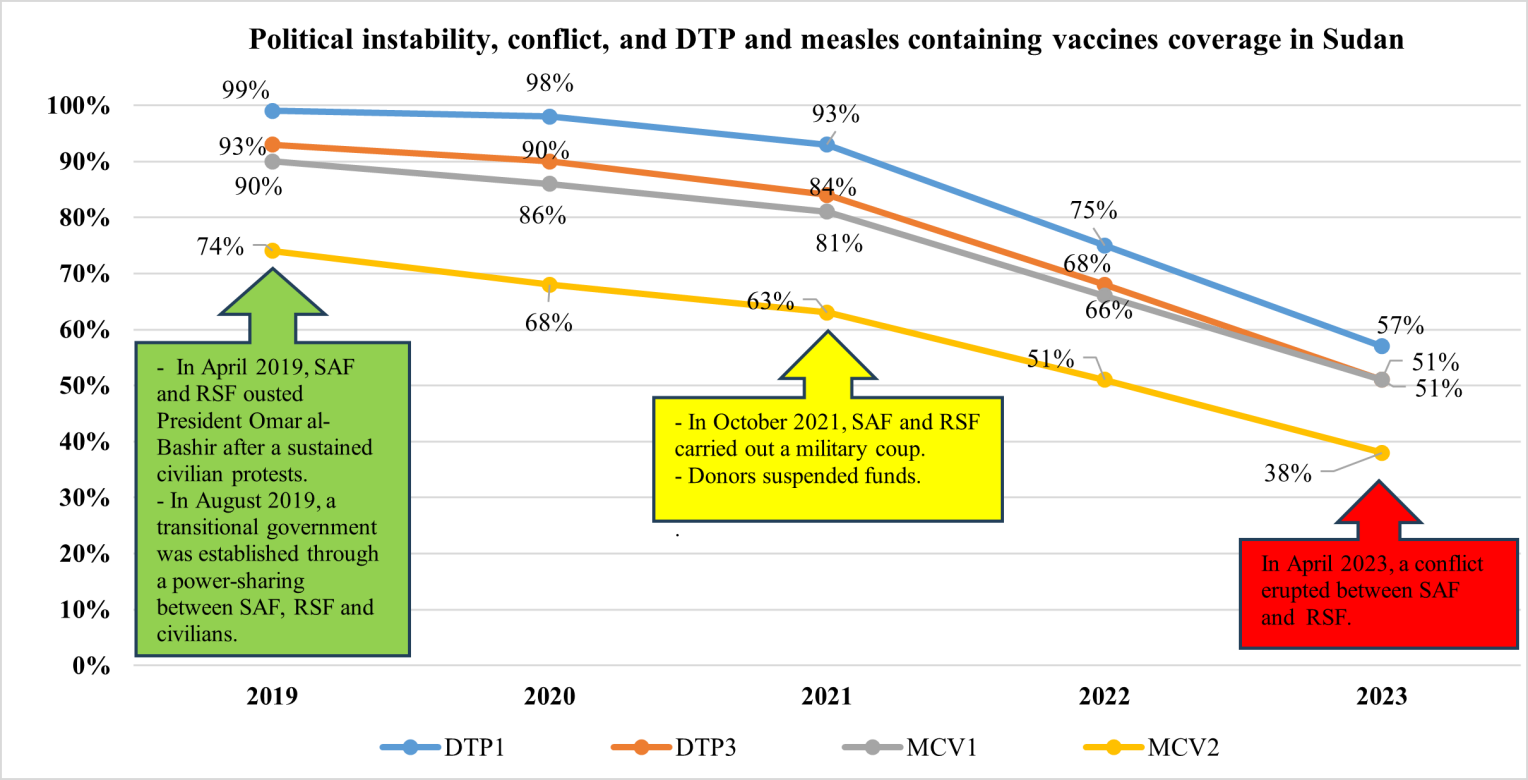
Political instability, conflict and DTP-containing vaccine and measles-containing vaccines coverage in Sudan. DTP, diphtheria, tetanus and pertussis; MCV, measles-containing vaccine; SAF, Sudanese Armed Forces; RSF, Rapid Support Forces.

Measles elimination requires achieving≥95% coverage with both measles-containing vaccine (MCV) doses to all children in every district. MCV coverage for the two doses is consistently below 90% in the five countries between 2019 and 2023, except for Burkina Faso. Sudan achieved 90% coverage for MCV1 in 2019, but there were 3555 cases of measles in the same year with a sharp drop of MCV1 and MCV2 in 2023 (51% and 38%, respectively); demonstrated in figure 2. Within this suboptimal coverage, there is considerable variability across different countries. Burkina Faso has maintained a relatively higher MCV1 coverage rate, reporting 88% coverage over 3 years and increased to 91% and 94% in 2022 and 2023, respectively. On the other hand, Chad has had alarmingly low coverage rates over these five years, which increased slowly from 41% in 2019 to 63% in 2023.

Measles vaccination coverage gaps are a common challenge across these countries, as MCV2 coverage is lower compared with MCV1. Chad’s MCV2 coverage is very low, at just 35% in 2023; however, it increased from 2% in 2022 when it was first introduced. Mali has made progress in this area; since introducing MCV2 in 2019, its coverage rates significantly increased from 4% to 59% by 2023. These low coverage rates across the five countries underscore the persistent risk of measles.

### Outbreaks

#### Polio (cVDPV)

The trend of polio outbreaks (cVDPV) across these five countries (2019–2023) showed fluctuations in the number of cases. Niger has consistently reported outbreaks over the past 5 years, with an increase in recent years from 10 cases in 2019 to 18 and 15 cases of polio (cVDPV) in 2021 and 2022, respectively, and dropped to 3 cases in 2023. Chad had the highest number of polio cases, with a total of 211 cases, with 101 and 55 cases reported in 2020 and 2023.

Despite relatively high estimated vaccination coverage, there were significant outbreaks of polio in Burkina Faso (65 cases), Sudan (59 cases) and Mali (52 cases) in 2020.

Genomic testing showed there is a link between the outbreaks of emergent variants across these countries. For example, the CHA-NDJ-1 variant was first detected in Chad in 2019 and then spread to other countries, including Sudan. Another genetic link has been established between the variants, especially NIE-JIS-1 was first detected in Nigeria in 2018 and then has been detected in other countries, including Burkina Faso, Chad, Mali and Niger.^42^

#### Measles

The scale of measles outbreaks and the number of cases reported varies significantly among these countries and over a 5-year period. For instance, Niger experienced a significant number of cases over the 5 years, with a dramatic spike of 12 898 cases reported in 2022. In Chad, the number of measles cases peaked at 11 862 in 2023. In light of MCV1 and MCV2 coverage in the five countries remaining significantly below the required 95%, the risk of measles outbreaks alarmingly persists.

It was noticed that there was missing data regarding measles cases in the WHO dashboard for 2 years (2021–2022) for Sudan and one year (2021) for Burkina Faso, which may indicate a breakdown in monitoring mechanisms. However, during a period of 7 months (April–November 2023) of active war in Sudan, 4413 suspected cases were reported, with 108 deaths. It is important to note that over 70% of the health facilities are no longer operational, and 65% of the population has no access to healthcare in conflict-affected states, which may suggest under-reporting of measles cases, especially during the active conflict.^23^

#### Other major vaccine-preventable disease outbreaks in the region

There are ongoing outbreaks of diphtheria, which began in 2023 in four African countries, mainly in West Africa: Guinea, Niger, Nigeria and Algeria.^43^ As of May 2024, a total of 27 511 were confirmed, with 1174 deaths reported across seven countries since 2023: Cameroon, Gabon, Guinea, Nigeria, Niger, Mauritania and South Africa. Nigeria alone accounts for over 78% of these cases, while Niger reported 15% of them.^43^

#### The added impact of the COVID-19 pandemic

In January 2020, following WHO’s declaration of COVID-19 as a Public Health Emergency of International Concern, the Global Polio Eradication Initiative shifted its focus towards mitigating the pandemic.^44^ Consequently, the programme temporarily halted supplementary immunisation activities (SIAs), while maintaining crucial poliovirus surveillance functions. This immediately resulted in the suspension of 62 polio vaccine SIAs in 28 countries worldwide.^44^

The impact of the COVID-19 pandemic on vaccination programmes in these five countries is not well documented, showing inconsistency between the vaccination coverage data and the number of VPD cases. For instance, in Burkina Faso, the vaccination coverage for all vaccines remained consistent over the four years. However, the number of cVDPV cases increased from 1 in 2019 to 65 cases in 2020, along with a fourfold increase in measles cases during the same period. Vaccination coverage in Chad showed an increasing trend. For example, DTP1 coverage increased from 65% in 2019 to 69% in 2020, and MCV1 from 41% to 47% during the same period. Despite this relative improvement, both these coverage rates remain suboptimal, while the number of polio (cVDPV) cases also increased from 11 cases in 2019 to 101 cases in 2020. During the same period, however, Sudan and Mali experienced a slight decline in vaccination coverage, coupled with polio (cVDPV) outbreaks in 2020, with 59 and 52 cases reported, respectively.

#### Case study: Sudan – political instability, conflict and declining vaccination coverage

The potential impact of political instability and ongoing conflict on vaccination coverage in Sudan is demonstrated in figure 2. In April 2019, sustained civilian protests led to the ousting of President Omer al-Bashir by the Sudanese Armed Forces (SAF) and Rapid Support Forces (RSF). A transitional government was established in August 2019 through a power-sharing agreement with civilian representatives. However, in October 2021, the SAF and RSF executed a military coup, halting the democratic transition.^45^ Before the coup d’état, Sudan had a relatively high vaccination coverage. For example, the first dose of the pentavalent vaccine (ie, DTP-containing vaccine) coverage, was 98% and 93% in 2020 and 2021, respectively. However, in 2022, the year after the coup d’état, the DTP1 coverage was 75%. One of the challenges during that year was a restriction on funding from international donors following the coup.^27^

In April 2023, conflict broke out between the SAF and the RSF, resulting in a drop in DTP-containing vaccine coverage to 57%.^6^ The second dose of the DTP-containing vaccine and the two doses of measles vaccination coverage also decreased significantly during the same period. Sudan is ranked sixth out of 10 countries with the highest number of zero-dose children and fifth out of 10 countries with the lowest DTP-containing vaccine coverage worldwide.^6^ Prior to this 2023 ranking, Sudan had never been on this list.

Nearly 25 million people in Sudan, almost half the population, require humanitarian assistance, including childhood immunisation and other health services.^12^ This situation is expected to significantly increase the number of cases and deaths from vaccine-preventable diseases among children in those areas.

### Recommendations

#### Stronger data to identify gaps and shape responses

This paper underscores significant challenges regarding the quality of vaccination coverage and surveillance data, and a lack of targeted analysis of conflict-affected populations in the Sahel. Furthermore, there is a significant reliance on census projections for calculating vaccination coverage, which is not reliable in crisis-affected settings.^46^

Using advanced methods such as geographical information systems could improve the estimate of vaccination coverage in conflict-affected areas where data are under-representative or unavailable. For example, the model-based geostatistical method was used in northern Nigeria to provide alternative vaccine coverage estimates for areas where traditional surveys are impractical due to conflict.^47^

Lessons can be learned from other relevant sectors, such as nutrition and food security, including through globally coordinated research programmes that focus on improving data collection for estimating vaccination coverage.^46^ For instance, the Semi-Quantitative Evaluation of Access and Coverage, a coverage assessment method for severe acute malnutrition resulted from a collaboration between various NGOs, UNICEF and academic institutions.^48^ A stronger culture of data sharing among agencies and prompt publishing of relevant information will further strengthen insights.

#### Centring childhood vaccination within political structures

Successful use of diplomatic approaches for vaccination access has been documented through negotiated ‘Days of Tranquillity’ and through collaboration with security forces.^49^ A diplomatic approach, for example, contributed to the ending of the 11-month boycott of the polio vaccine in the northern states of Nigeria in 2003, a region with a history of political, ethnic and religious tensions.^31^ It is worth noting, however, the inherent power imbalances that are entangled within these approaches.

#### Investment in innovative and flexible approaches at the community level

The integration of vaccines with other essential health services may help maximise the impact of health interventions and use every contact with the healthcare system in stable areas as an opportunity to vaccinate. Evidence showed that using these strategies has increased vaccination coverage and reduced missed opportunities for vaccination in similar fragile and conflict contexts in Nigeria, Cameroon, South Sudan and Somalia.^50^

Greater investment in civil society and community-based organisations can strengthen innovative strategies for co-delivery of vaccines in inaccessible areas. Lessons can be taken from successful local initiatives and best practices such as Gavi funded, RAISE 4 Sahel and REACH. These initiatives were implemented by NGOs^37^ to reach zero-dose children in non-state-controlled areas. In addition to increasing access, these support trust-building efforts with community groups in conflict-affected areas.

Ideally, vaccination should be offered as part of comprehensive maternal and child healthcare in primary healthcare services. However, in fragmented health systems and crisis contexts, these services are heavily affected by insecurity, humanitarian challenges and mistrust. Addressing contextual and programmatic challenges through innovative and flexible approaches is critical if zero-dose and underimmunised children in the Sahel are to be reached.

## Data Availability

Data are available in a public, open access repository.
